# Mild behavioral impairment in Parkinson's disease is associated with altered corticostriatal connectivity

**DOI:** 10.1016/j.nicl.2020.102252

**Published:** 2020-03-27

**Authors:** Stefan Lang, Eun Jin Yoon, Mekale Kibreab, Iris Kathol, Jenelle Cheetham, Tracy Hammer, Justyna Sarna, Zahinoor Ismail, Oury Monchi

**Affiliations:** a Cumming School of Medicine, University of Calgary, Calgary, AB, Canada; b Department of Clinical Neuroscience, University of Calgary, Calgary, AB, Canada; c Hotchkiss Brain Institute, University of Calgary, Calgary, AB, Canada; d Department of Psychiatry, University of Calgary, Calgary, AB, Canada; e Mathison Center for Brain and Mental Health Research, University of Calgary, Calgary, Canada; f Department of Radiology, University of Calgary, Calgary, AB, Canada

**Keywords:** Parkinson's disease, Cognition, Mild behavioral impairment, Corticostriatal connectivity, Resting state, Basal ganglia, Default mode network

## Abstract

**Background:**

Mild behavioral impairment (MBI) is a syndrome characterized by later life onset, sustained neuropsychiatric symptoms as a marker of dementia risk. In Parkinson's disease (PD), MBI has been associated with worse cognitive abilities and increased cortical atrophy. However, the circuit level correlates of MBI have not been investigated in this population. Our objective was to investigate the relationship between MBI and corticostriatal connectivity in PD patients. This emphasis on corticostriatal connectivity was due to the significant role of these circuits in neuropsychiatric and cognitive symptoms across disease conditions.

**Methods:**

Seventy-four non-demented patients with PD were administered the MBI-checklist, and classified as having high MBI (PD-MBI; *n* = 21) or low MBI scores (PD-noMBI; *n* = 53). Corticostriatal connectivity was assessed with both an atlas and seed-based analysis. The atlas analysis consisted of calculating the average connectivity between the striatal network and the default mode (DMN), central executive (CEN), and saliency networks (SAN). Structural measurements of cortical thickness and volume were also assessed. PD-MBI and PD-noMBI patients were compared, along with a group of age matched healthy control subjects (HC; *n* = 28). Subsequently, a seed analysis assessed the relationship of MBI scores with the connectivity of twelve seeds within the striatum while controlling for cognitive ability. A complementary analysis assessed the relationship between striatal connectivity and cognition, while controlling for MBI-C.

**Results:**

PD-MBI demonstrated decreased connectivity between the striatum and both the DMN and SAN compared to PD-noMBI and HC. The decreased connectivity between the striatum and the SAN was explained partly by increased atrophy within the SAN in PD-MBI. The seed analysis revealed a relationship between higher MBI scores and lower connectivity of the left caudate head to the dorsal anterior cingulate cortex and left middle frontal gyrus. Higher MBI-C scores were also related to decreased connectivity of the right caudate head with the anterior cingulate cortex, precuneus, and left supramarginal gyrus, as well as increased connectivity to the left hippocampus and right cerebellar hemisphere. Caudate-precuneus connectivity was independently associated with both global behavioural and cognitive scores.

**Conclusion:**

These results suggest PD-MBI is associated with altered corticostriatal connectivity, particularly between the head of the caudate and cortical regions associated with the DMN and SAN. In particular, caudate-precuneus connectivity is associated with both global behavioral and cognitive symptoms in PD.

## Highlights

• Mild behavioral impairment in PD is linked to altered corticostriatal connectivity.
• PD-MBI have less connectivity between the striatum and the DMN.
• PD-MBI have increased atrophy of the SAN.
• Caudate head and dorsal putamen connectivity is related to MBI-C scores in PD.
• Caudate head-precuneus connectivity is linked to both MBI and MoCA scores.

PD: Parkinson's disease
HC: healthy control
CEN: central executive network
SAN: saliency network
DMN: default mode network
MCI: mild cognitive impairment
MBI: mild behavioral impairment
MBI-C: mild behavioral impairment checklist
PD-MBI: Parkinson's disease with high MBI-C scores
PD-noMBI: Parkinson's disease with low MBI-C scores
NPI: neuropsychiatric inventory
NPS: neuropsychiatric symptoms

## 1 Introduction

Patients with Parkinson's disease suffer from many non-motor symptoms, which greatly impact quality of life (Aarsland et al., 2009; Foltynie et al., 2004; Schrag et al., 2000). Non-motor symptoms include cognitive impairment, as well as behavioral symptoms of depression, anxiety, apathy, impulse control, perceptual dysfunction, and disorders of thought (Poewe, 2008; Schapira et al., 2017). Collectively, these behavioral non-motor phenomenon are known as neuropsychiatric symptoms (NPS) (Aarsland et al., 2009; Kulisevsky et al., 2008). NPS are among the most common non-motor symptoms in Parkinson's disease (Aarsland et al., 2009; Schapira et al., 2017), can affect patients even in prodromal stages (Tolosa et al., 2007), and are frequently comorbid with cognitive impairment and dementia (Aarsland et al., 2014). Importantly, NPS in PD can predict cognitive decline (Pirogovsky-Turk et al., 2017), suggesting that these symptoms may be early warning signs of dementia.

Mild behavioral impairment (MBI) is a validated syndrome that captures later life acquired (onset after 50 years old), sustained (>6 months) NPS which are considered an at-risk state for incident cognitive decline and dementia, and the index manifestation of dementia for some (Ismail et al., 2016). MBI is distinct from chronic and recurrent psychiatric disorders. A five year longitudinal study demonstrated that older adults with MBI have a higher conversion rate to dementia compared to a group of individuals with late life recurrent psychiatric conditions (Taragano et al., 2018). Similarly, a study in an outpatient psychiatric clinic demonstrated higher rate of incident dementia in MBI versus other psychiatric conditions (Matsuoka et al., 2019). MBI has been associated with worse cognitive abilities at baseline, is a marker of subsequent cognitive decline in cognitively normal older adults (Creese et al., 2019a), and has been shown to increase the rate of progression to dementia compared to those with mild cognitive impairment (MCI) (Taragano et al., 2009). MBI thus represents the neurobehavioral axis of predementia risk states. The MBI checklist (MBI-C) has been developed to explicitly measure MBI, and consists of a 34 item instrument which is easily completed by a patient, close informant, or clinician (Ismail et al., 2017). The MBI-C was structured to be consistent with the five domains of MBI: decreased motivation, emotional dysregulation, impulse dyscontrol, social inappropriateness, and abnormal perception or thought content. The MBI-C has been validated, and generates both domain specific scores and an overall score, which can be used to classify individuals as having MBI or not (Creese et al., 2019b; Hu et al., 2019; Mallo et al., 2019, 2018).

While the assessment of neuropsychiatric symptoms in PD is common, the specific evaluation of MBI has been limited. A major difference between the MBI construct and the more commonly used index of NPS (the Neuropsychiatric Inventory; NPI), is the requirement of late life acquired and sustained symptoms in MBI. Importantly, MBI excludes psychiatric illness a priori, which may allow for differentiation from co-morbid psychiatric disease that are independent from the neurodegenerative process (Ismail et al., 2017). One recent study assessed MBI in PD by using the NPI with a modified reference range of six months, and by computing MBI domains using NPI sub-scores (Baschi et al., 2019). The frequency of MBI was 84.1% throughout the entire PD sample, and 36.1% in the newly diagnosed group. In this study, there was no relationship between MBI status and mild cognitive impairment (MCI) status after controlling for age, sex, and education. Multivariate analysis revealed an association between MBI and antidepressant use in newly diagnosed PD, and MBI and motor severity in late PD (Baschi et al., 2019). However, continuous relationships between MBI scores and cognitive scores were not assessed, and MBI status was determined with a cut-off of just one behavioral symptom across all 12 NPI domains, which may not provide adequate specificity. In contrast, recent data from our group using the MBI-C, which was developed as a case ascertainment instrument specifically for MBI, suggests a strong relationship between MBI scores and cognitive ability when evaluated in a continuous manner (Yoon et al., 2019). Further, when a higher cut-off was used, there was a significant relationship between MBI and MCI status (Yoon et al., 2019). This is consistent with previous literature showing significantly more NPS in PD-MCI vs PD-nonMCI (Monastero et al., 2013). Overall, we believe the sum of evidence supports a relationship between MBI and cognition, suggesting these phenomena have an overlapping neural representation.

One strong candidate for a shared neural substrate between global behavioral and cognitive symptoms across neurodegenerative diseases is dysfunction of corticostriatal networks (O'Callaghan et al., 2014). Corticostriatal networks have been widely implicated in the cognitive impairment experienced by patients with Parkinson's disease (Hanganu et al., 2015; Leh et al., 2009; Seibert et al., 2012). Further, the striatum and corticostriatal networks have been implicated in several of the individual NPS experienced by patients with PD (for example: anxiety (Erro et al., 2012; Oosterwijk et al., 2018), depression (Vriend et al., 2014), and apathy (Baggio et al., 2015a; Santangelo et al., 2018), for a review see Valli et al. (2019) and Wen et al. (2016). The mechanisms of striatal and corticostriatal network dysfunction which gives rise to global NPS are likely related to degeneration of various neuromodulatory systems, including the dopaminergic, serotonergic and noradrenergic systems (Castrioto et al., 2016; Ceravolo et al., 2013; Maillet et al., 2016).

Our objective was to investigate the relationship between corticostriatal connectivity and MBI (as measured by the global score of the MBI-C) in PD. We compared corticostriatal connectivity in PD-MBI vs PD-noMBI and healthy controls. Given the relationship between NPS and cognitive symptoms, we hypothesized that altered connectivity would be observed between the striatum and cortical networks implicated in both psychopathology (Bressler and Menon, 2010; Menon, 2011) and cognitive impairment (Baggio et al., 2015b; Biundo et al., 2016). This was investigated with an atlas-based analysis, looking at the connectivity between the striatal network and the default mode (DMN), central executive (CEN), and saliency networks (SAN). We then directly assessed the relationship between connectivity of subregions of the striatum to MBI-C scores using a seed-based analysis. Finally, we investigated whether there was some overlap in striatal connectivity patterns related to both global behavioral and cognitive impairment.

## 2 Methods

### 2.1 Subjects

Seventy-four non-demented Parkinson's disease subjects at stages I – III of Hoehn and Yahr were diagnosed by movement disorder neurologists and met the UK brain bank criteria for idiopathic Parkinson's disease (Hughes et al., 1992). Subjects were recruited from the Movement Disorder Clinic at the University of Calgary between 2014 and 2019. Exclusion criteria included neurological disease aside from idiopathic PD, dementia, inability to tolerate MRI scans, or previous deep brain stimulation surgery. In particular, subjects with severe psychiatric disease (including alcohol or drug dependency) documented in their clinical medical records by a physician were excluded from this study. Subjects underwent a comprehensive cognitive assessment and had MRI scans administered (within the same week). All subjects were asked to continue taking their regular scheduled medications. The MBI-C was completed by the informant (family member or caregiver). A control group, twenty-eight age-matched healthy subjects underwent the same protocol including the MBI-C. Controls were excluded if they had a neurological or psychiatric diagnosis, if they met criteria for MCI, or if they had a blood relative with PD. These subjects were recruited through recruitment flyers placed in the hospital and university environment. All subjects provided informed consent, and the protocol was approved by the University of Calgary Research Ethics Board. Subject demographic characteristics are listed in Table 1.

**Table 1:** Demographic table.

						Post-Hoc		
	PD-MBI	PD-noMBI	HC	Test statistic (F/H/t/U/x^2^)	p-value	PD-MBI vs PD-noMBI	PD-MBI vs HC	PD-noMBI vs HC
N	21	53	28	–	–	–	–	–
Age	71.8 +/−6.4	70.4 +/- 5.8	69.8 +/- 6.7	*F* = 0.67	0.52	–	–	–
Gender (F:M)	6:15	19:34	15:13	x^2^ = 3.67	0.16	–	–	–
Education (years)	13.9 +/- 3.3	15.2 +/- 2.5	16.2 +/- 2.8	*H* = 6.99	0.03	–	*	–
UPDRS-III	22.9 +/- 8.0	17.2 +/- 11.0	–	*t* = −2.17	0.03	–	–	–
LED (mg/day)	834.0 +/−412.0	742.1 +/- 369.9	–	*t* = −0.93	0.35	–	–	–
Disease Onset (age/years)	66.16 +/- 7.98	64.83 +/- 6.46	–	*t* = −0.75	0.46	–	–	–
Disease Duration (years)	5.68 +/- 3.75	5.55 +/- 3.99	–	*t* = −0.12	0.90	–	–	–
MoCA	22.8 +/- 4.3	26.0 +/- 3.5	27.2 +/- 2.1	*H* = 13.8	0.001	**	***	ns
Executive Function	−0.65 +/- 0.82	−0.20 +/- 0.80	0.11 +/- 0.47	*H* = 10.5	0.005	ns	**	ns
Attention	−0.50 +/−0.58	−0.14 +/- 0.57	0.28 +/- 0.46	*F* = 12.5	<0.0001	*	***	**
Language	−0.45 +/- 0.67	0.00 +/- 0.76	0.40 +/- 0.87	*F* = 7.1	0.001	ns	***	ns
Visuospatial	−0.63 +/- 0.88	−0.21 +/- 0.92	0.29 +/- 0.35	*H* = 17.5	0.0002	*	***	*
Memory	−0.62 +/- 0.92	0.12 +/- 0.70	0.27 +/- 0.38	*H* = 12.3	0.0021	**	**	ns
MCI:No MCI	13:8	19:34	–	x^2^ = 4.16	0.041	–	–	–
MBI-C (Total)	15.4 +/- 9.8	1.68 +/- 2.0	0.39 +/- 1.4	*H* = 63.1	<0.0001	***	***	*
Drive/Motivation	3.62 +/- 3.60	0.47 +/- 0.82	0.07 +/- 0.38	*H* = 42.3	<0.0001	***	***	ns
Mood/Anxiety	5.14 +/- 3.2	0.51 +/- 1.1	0.11 +/- 0.42	*H* = 63.9	<0.0001	***	***	ns
Impulse dyscontrol	4.80 +/- 5.4	0.47 +/- 0.91	0.21 +/- 0.83	*H* = 42.2	<0.0001	***	***	ns
Social Inappropriateness	1.24 +/- 2.2	0.06 +/- 0.23	0 0.0 +/- 0.0	*H* = 30.6	<0.0001	***	***	ns
Perception/Thought	0.62 +/- 0.97	0.17 +/- 0.61	0 0.0 +/- 0.0	*H* = 12.7	0.002	*	**	ns
Mean Movement (mm/TR)	0.19 +/- 0.10	0.22 +/- 0.14	0.17 +/- 0.07	*H* = 2.04	0.361	–	–	–

UPDRS: Unified Parkinson Disease Rating Scale; LED: Levodopa Equivalent Dose; MoCA: Montreal Cognitive Assessment.

All variables reported as mean +/- standard deviation.

Cognitive scores reported as z-scores.

*F = one-way ANOVA; H = Kruskal-Wallis; x^2^ = chi-squared; t = two sample t-test*.

ns = post-hoc p > 0.05; * = post hoc p < 0.05; **=post hoc p < 0.01; ***=post-hoc p < 0.001.

### 2.2 Neuropsychological assessment

All subjects underwent a comprehensive neuropsychological evaluation with a total of 15 tests to assess five cognitive domains (attention, executive functioning, language, memory and visuospatial ability) (Supplementary Table 1). The MoCA was also administered as a test of global cognitive function. Each test was administered and scored by a psychometrist, and the raw scores were converted to z-scores based on appropriate normed data. Subsequently, domain specific average z-scores were calculated. Subjects were classified as having MCI if they met the Movement Disorder Task Force Level II criteria for MCI in Parkinson's disease (Litvan et al., 2012). These requirements were as follows: (1) performance >1.5 SD below the standardized mean on at least 2 tests within or across cognitive domains; (2) subjective complaint of cognitive decline by patient or accompanying person; (3) absence of significant decline in daily living activities; (4) absence of dementia. Healthy controls underwent the same neuropsychological evaluation, and were excluded if they met the criteria for MCI.

### 2.3 MBI assessment

The MBI-C was completed by a suitable informant, in accordance with MBI validation studies (Ismail et al., 2017; Kang et al., 2018). This questionnaire consists of 34 items organized into 5 MBI domains (Drive/Motivation; Mood/Anxiety; Impulse dyscontrol; Social inappropriateness; Perception/Thought). For each item, a ‘yes’ or ‘no’ question is followed by a severity rating scale of 1 (mild), 2 (moderate), or 3 (severe). Each symptom must be present for at least 6 months and represent a meaningful change from baseline. Consistent with previous literature (Mallo et al., 2019, 2018), including work from our group (Yoon et al., 2019), we dichotomized the PD patients into PD-MBI or PD-noMBI using a cut-off score of 7.5. No HC reached this threshold.

### 2.4 MRI acquisition and image pre-processing

Subjects were scanned at the Seaman Family MR Center, at the University of Calgary, with a 3T GE Discovery MR750 scanner. Sessions included a high-resolution, T1-weighted, 3D volume acquisition followed by echo-planar T2*-weighted image acquisitions with BOLD contrast for resting-state analysis. Images were pre-processed and denoised in a fashion consistent with our previous work (Lang et al., 2019), using SPM 12 (Friston, 2007) and the Conn toolbox (Whitfield-Gabrieli and Nieto-Castanon, 2012). Briefly, functional images underwent realignment and unwarping as well as slice-time correction, prior to non-linear normalization into MNI space. Physiological and other sources of noise from the white matter and CSF signal were estimated using the aCompcor method (Behzadi et al., 2007; Chai et al., 2012). To account for motion, movement parameters, and their first temporal derivative, were also included in the regression. Full details can be found in the Supplementary Methods I.

### 2.5 Atlas-based analysis

**Fig. 1:**
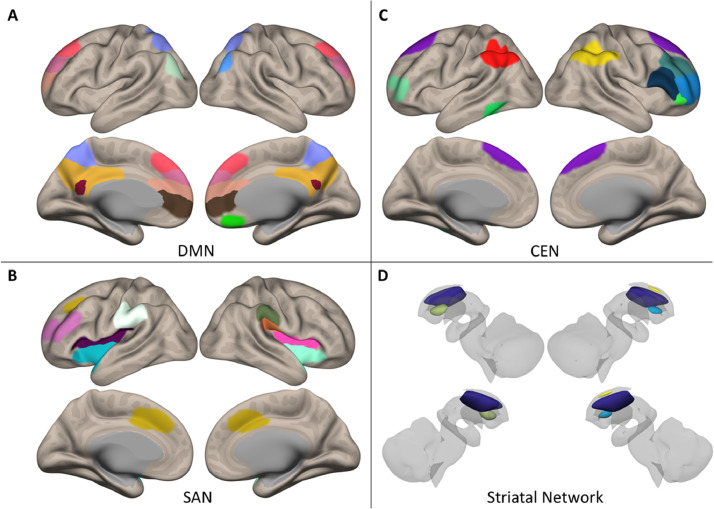
Regions of interest for Atlas-based analysis. A) Default mode network (DMN); B) Central executive network (CEN); C) Saliency network (SAN); D) Striatal network. All networks adopted from (Lang et al., 2019).

Cortical and subcortical networks were selected from our previous work (Lang et al., 2019). These networks were originally defined through high dimensional group ICA (Calhoun et al., 2001), followed by assessing each component's spatial similarity with the Stanford Functional Atlas (Shirer et al., 2012). In the present manuscript, we assessed connectivity between the striatal network and the DMN, the CEN, and the SAN (Fig. 1). ROI details can be found in Supplementary Table 2.

The residual BOLD time course was averaged amongst voxels within each individual ROI. As a standard measure of functional connectivity, the Pearson Correlation Coefficient was calculated between each ROI. To improve normality of the correlation measure, a Fisher transformation was applied. Internetwork connectivity strength is defined by the mean connection strength of each ROI pair between networks using the *conn_withinbetweenROItest* function implemented in the Conn toolbox. In this manner, a value representing the connectivity between the striatal network and each cortical network (DMN, CEN, SAN) was obtained for every subject. To determine whether any group differences in functional connectivity were being driven by differences in structural measures, we calculated the average thickness (for each cortical network) and the average volume (for the striatal network). The details of the structural analysis can be found in Supplementary Methods II.

### 2.6 Seed based analysis

**Fig. 2:**
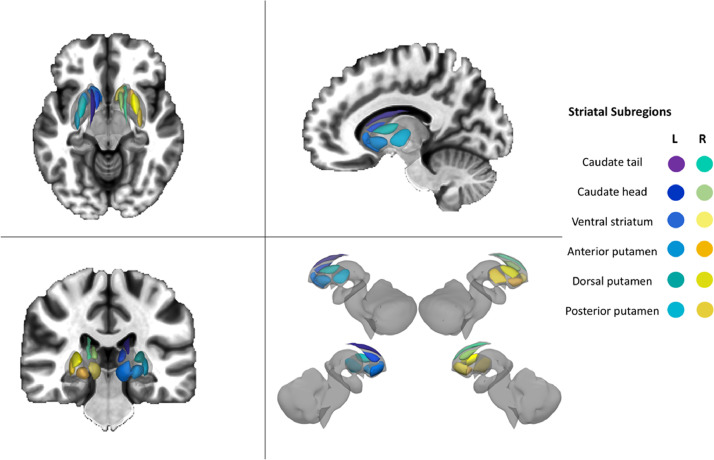
Striatal subdivisions for seed-based analysis, adopted from a model-based functional parcellation (Janssen et al., 2015). This parcellation included 12 regions of interest (six per hemisphere): (1) caudate tail, (2) caudate head, (3) ventral striatum, (4) anterior putamen, (5) dorsal putamen, and (6) posterior putamen.

To gain finer spatial resolution regarding the relationship between corticostriatal connectivity and MBI-C scores, we performed a seed-based analysis with twelve regions of interest (six per hemisphere) located throughout the basal ganglia. These were adopted from a model-based functional parcellation (Janssen et al., 2015) of the striatum and included: the dorsal caudate/caudate tail, ventral caudate/caudate head, ventral striatum/nucleus accumbens, anterior putamen, dorsal putamen, and posterior putamen (Fig. 2). The residual BOLD time course was averaged within each striatal ROI, followed by evaluation of the Fisher Z transformed Pearson correlation with the BOLD time course from every other voxel within the brain. In this manner, whole brain connectivity maps for each striatal ROI were obtained for each subject. When appropriate, we also measured the relationship between the volume of each of these striatal regions and MBI-C scores (Supplementary Methods II).

### 2.7 Statistical analysis

All statistical analyses were performed in MATLAB (MathWorks®, MA, USA). Kolmogorov-Smirnov tests were used to assess for normality of the data, while Levene's test was used to assess for equality of variance. Demographic variables were compared between PD-MBI, PD-noMBI, and healthy control subjects with a combination of one-way ANOVA, Kruskall-Wallis, and chi-squared tests. When appropriate, post-hoc testing was performed with Tukey or Mann-Whitney U tests. Group differences in network connectivity between PD-MBI, PD-noMBI, and HC were evaluated with Welch's ANOVA and post-hoc Games-Howell tests. These are robust to heteroscedasticity and unequal sample sizes (Games and Howell, 1976; Welch, 1947). The effect of education was adjusted for in this analysis, given the significant group difference in this demographic variable. Significance for the main effect of group was set at *p* < 0.0167 to correct for 3 internetwork calculations. When appropriate, post-hoc comparison of means was performed with Games-Howell Tests, and significance was set at *p* < 0.05. We repeated post-hoc comparisons between PD-MBI and PD-noMBI after adjustment of the Unified Parkinson's Disease Rating Scale part III scores (UPDRS-III), to ensure this variable was not responsible for differences in connectivity.

For the seed-based analysis, each subjects’ first level connectivity map (for each striatal ROI) was used as input for a second level analysis assessing the relationship of voxel-wide connectivity and MBI-C scores within the PD group. This was implemented in Conn using the general linear model and the likelihood ratio test to evaluate model parameters. Significant clusters were defined with a height threshold of *p* < 0.001 (two-tailed, uncorrected), followed by a cluster threshold of *p* < 0.05 with a false discovery rate (FDR) correction for multiple comparisons. We included UPDRS-III and MoCA scores as covariates of no interest in this analysis, thereby adjusting for motor function and global cognitive ability. Significant clusters therefore represented the effect of MBI-C independent from global cognition and motor severity. In a subsequent analysis, we aimed to examine if there was overlap between the relationship of corticostriatal connectivity with behavioural and cognitive abilities. To this end, we repeated the above analysis for striatal subregions showing a significant effect of MBI-C, but now assessing for the effect of MoCA while adjusting for MBI-C and UPDRS-III. Analysis of the significant seeds were repeated after the quantitative assessment, and removal, of any outliers (Supplementary Methods III).

### 2.8 Data availability

The data that support the finding of this manuscript are available from the corresponding author, upon request.

## 3 Results

### 3.1 Demographic variables

All demographic variables, including group comparisons, are displayed in Table 1. There was no difference between groups in age or gender. The PD-MBI group had significantly less education as compared to the HC group (H(2,99) = 6.99, *p* = 0.03, post-hoc *p* = 0.023). There was no difference in education between PD-noMBI and HC, nor between PD-noMBI and PD-MBI. Significantly higher UPDRS-III scores were observed in the PD-MBI group as compared to the PD-noMBI (t(72) = −2.17, *p* = 0.03). However, there was no difference in overall levodopa usage or disease duration. All cognitive variables showed a significant group effect, with PD-MBI having poorer cognition in every domain compared to HC. PD-MBI also had lower scores in attention, visuospatial ability, memory, and MoCA as compared to PD-noMBI (Table 1). There were significantly more PD-MBI participants who were classified as MCI when compared to PD-noMBI. PD-noMBI had higher overall MBI-C scores compared to HC, but the difference was not significant for any of the individual MBI domains. There was no difference between groups in the amount of intra-scanner motion.

### 3.2 Atlas-based analysis

**Fig. 3:**
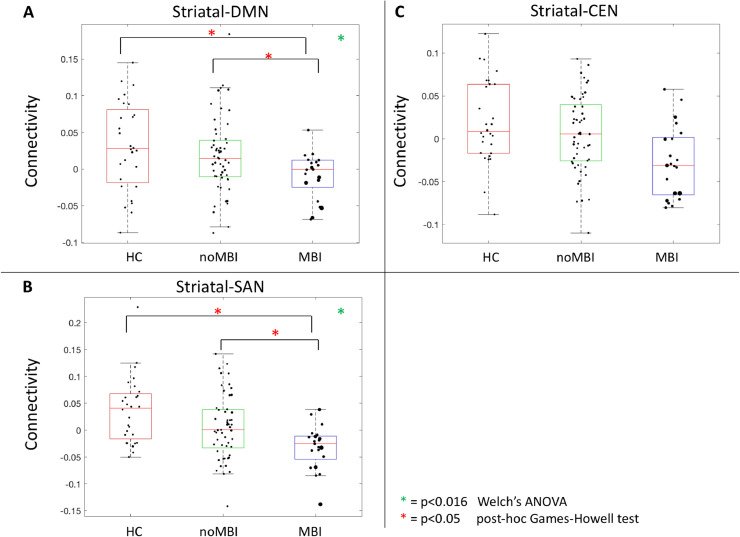
Group differences in Striatal-Cortical Network connectivity. A) Striatal network to DMN connectivity; B) Striatal network to SAN connectivity; C) Striatal network to CEN connectivity. Welch's ANOVA controlling for education with significance set at *p* < 0.0167. Post-hoc comparison using Games-Howell test with significance set at *p* < 0.05. All direct post-hoc comparisons between PD-MBI and PD-noMBI adjusted for the effect of UPDRS-III. For visualization, the diameter of each data point is representative of the MBI-C score: large data points equal higher scores.

Connectivity between the striatal network and three cortical networks (DMN, SAN, CEN) were compared between groups while adjusting for education. Direct post-hoc comparisons between PD-MBI and PD-noMBI were repeated while adjusting for UPDRS-III scores. Striatal to DMN connectivity was significantly different between groups (F(1,40.8)=7.66, *p* = 0.0085) (Fig. 3A). Post-hoc comparison demonstrated significant group differences between PD-MBI vs PD-noMBI (*p* = 0.023), as well as PD-MBI vs HC (*p* = 0.015), with lower connectivity in the PD-MBI group. There was no difference in striatal-DMN connectivity when comparing PD-noMBI vs HC (*p* = 0.674). Striatal to SAN connectivity also demonstrated a significant group difference (F(1,50.23)=6.60, *p* = 0.0132) (Fig. 3B). Post-hoc comparisons demonstrated lower connectivity in PD-MBI vs HC (*p* = 0.0023), and in PD-MBI vs PD-noMBI (*p* = 0.0354), but not in PD-noMBI vs HC (*p* = 0.1937). There were no group differences when assessing striatal to CEN connectivity (F(1,60.96) = 5.50, *p* = 0.0223), when corrected for multiple comparisons (Fig. 3C). When assessing the atrophy within the various networks, we observed a significant group difference in the average thickness of the SAN (F(1,43.75) = 7.74, *p* = 0.0079). Post-hoc tests revealed this was due to increased average atrophy of the SAN in PD-MBI vs PD-noMBI (*p* = 0.0221) and PD-MBI vs HC (*p* = 0.0039). There was no group difference in the atrophy pattern of the DMN (F(1,34.17)=1.56, *p* = 0.2204), the CEN (F(1,39.57)=0.060, *p* = 0.8072), or the striatal network (F(1,34.10)=1.96, *p* = 0.1706). Given the significant difference in atrophy within the SAN, we repeated the group comparison of functional connectivity between the striatal network and the SAN, while adjusting for average atrophy within the SAN. Once SAN atrophy was adjusted for, the group difference in functional connectivity was no longer statistically significant with correction for multiple comparison (F(1,52.40)=4.97, *p* = 0.0301) (Supplementary Methods II).

### 3.3 Seed-based analysis

**Fig. 4:**
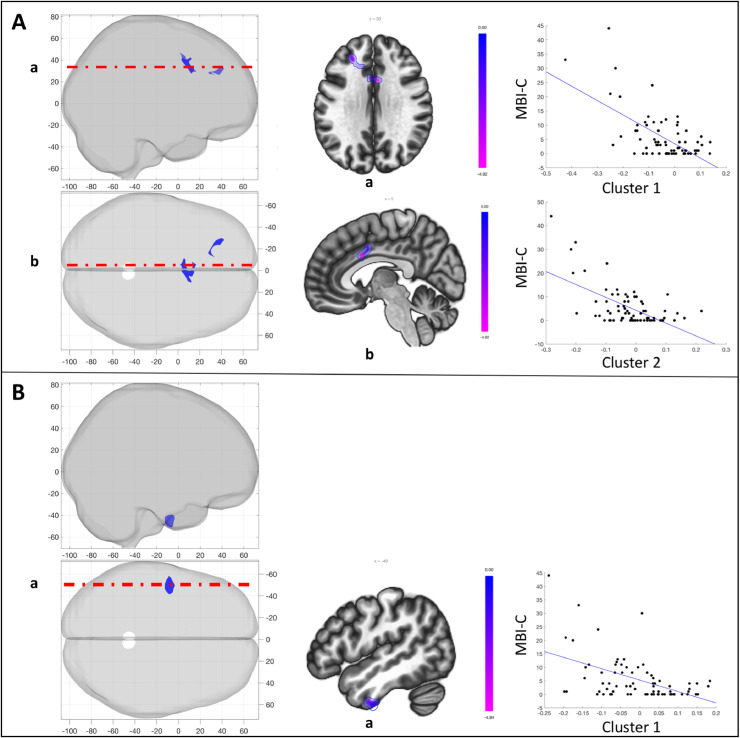
Relationship between MBI-C and striatal subdivision connectivity, adjusting for MoCA and UPDRS-III. A) Seed: Left caudate head. MBI-C was independently associated with left caudate head connectivity to the dorsal ACC (cluster 1) and the left MFG (cluster 2); B) Seed: Left dorsal putamen. MBI-C was independently associated with left dorsal putamen connectivity to the left ITG (cluster 1). Panels (left to right) represent: (1) 3-D volume rendering of significant clusters; (2) selected axial and sagittal slices for visualization; and (3) extracted relationship between MBI-C and connectivity for each cluster *(see*Table 2*for cluster details)*.

To gain finer spatial insight into the relationship of corticostriatal connectivity and MBI-C scores, the striatum was subdivided into 12 seeds. Connectivity between each one of these seeds and the whole brain was calculated for the PD group. Subsequently, striatal connectivity was related to MBI-C scores. Significant clusters were observed for the left caudate head, left dorsal putamen, and right caudate head seeds (*p* < 0.001, cluster *p* < 0.05 FDR; Table 2). Specifically, higher MBI-C scores were associated with decreased connectivity between the left caudate head and the dorsal anterior cingulate cortex and as well as between the left caudate head and the left middle frontal gyrus (Fig. 4A). Higher MBI-C scores were also associated with decreased connectivity between the left dorsal putamen and the left inferior temporal pole (Fig. 4B). One subject was deemed an outlier with respect to MBI-C scores. When removing this subject, MBI-C scores were associated with decreased connectivity between the left caudate head and the dorsal anterior cingulate cortex (Supplementary Methods III).

**Table 2:** Significant clusters from the seed-based analysis assessing the relationship of striatal connectivity with MBI-C scores. Analysis was adjusted for MoCA and UPDRS-III.

Seed	Location	MNI (x,y,z)	Size (voxels)	Peak p value	Cluster *p* value (FDR)
Left Caudate Head					
Cluster 1	Dorsal ACC	04, 12, 30	210	0.000006	0.0016
Cluster 2	Left MFG	−14, 30, 28	106	0.000015	0.0402
Left Dorsal Putamen					
Cluster 1	Left ITG	−58, −08, −42	166	0.000005	0.0031
Right Caudate Head					
Cluster 1	Precuneus/SOC	−14, −82, 48	631	<0.000001	<0.000001
Cluster 2	Dorsal ACC	12, 18, 42	410	0.000005	0.000002
Cluster 3	Left SMG/AG	−60, −44, 44	130	0.000010	0.0099
Cluster 4	Right preCG	16, −28, 56	114	0.000011	0.0142
Cluster 5	Left pHG	−30, −44, 00	105	0.000011	0.0167
Cluster 6	Right cerebellum	22, −72, −24	79	0.000020	0.0444

*ACC = anterior cingulate cortex; MFG = middle frontal gyrus; ITG = inferior tempral gyrus; SOC = superior occipital cortex; SMG = supramarginal gyrus; AG = angular gyrus; preCG = precentral gyrus; pHG = posterior hippocampal gyrus*.

*p < 0.001, cluster p < 0.05 FDR corrected.

**Fig. 5:**
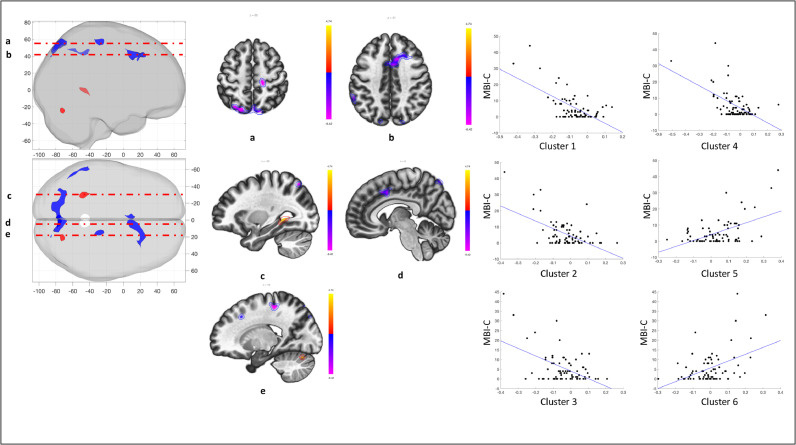
Relationship between MBI-C and right caudate head connectivity, adjusting for MoCA and UPDRS-III. MBI-C was independently associated with right caudate head connectivity to the precuneus/SOC (cluster 1), the dorsal ACC (cluster 2), left SMG/AG (cluster 3), right preCG (cluster 4), left pHG (cluster 5), and right cerebellar hemisphere (cluster 6). Panels (left to right) represent: (1) 3-D volume rendering of significant clusters; (2) selected axial and sagittal slices for visualization; and (3) extracted relationship between MBI-C and connectivity for each cluster *(see*Table 2*for cluster details)*.

The right caudate head showed a more widespread and variable relationship with MBI-C scores. Higher MBI-C scores were related to decreased connectivity between the right caudate head and the dorsal anterior cingulate cortex, a cluster spanning the precuneus and superior occipital cortex, a cluster spanning the left supramarginal and angular gyrus, and a cluster in the right precentral gyrus (Fig. 5). Higher MBI-C scores were also related to increased connectivity between the right caudate head and a cluster in the left posterior hippocampus and the right cerebellum (Fig. 5). With the outlier removed, clusters remained significant in the dorsal anterior cingulate, precuneus/superior occipital cortex, and right cerebellum. Further, a cluster emerged in the right middle/superior frontal gyrus. No significant clusters emerged from any of the remaining striatal seeds. Also, there was no significant relationship between MBI-C scores and regional volume in the significant seeds (left caudate head, left dorsal putamen, or right caudate head).

**Fig. 6:**
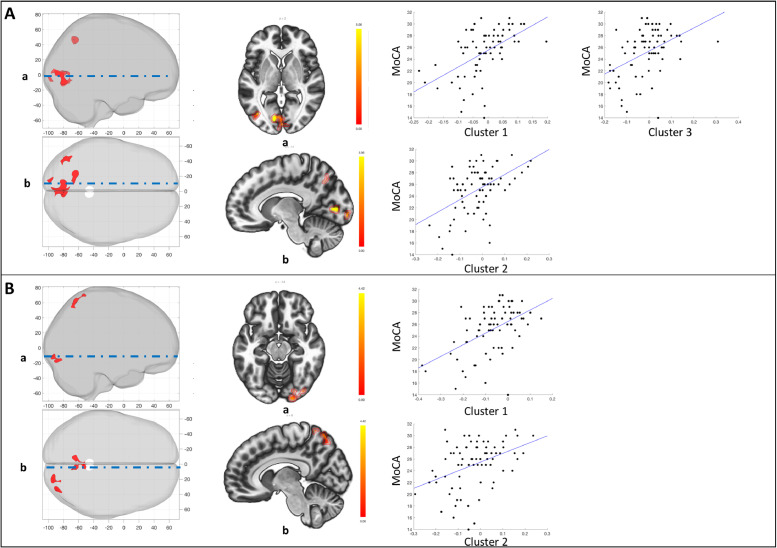
Relationship between MoCA and striatal subdivision connectivity, adjusting for MBI-C and UPDRS-III. A) Seed: Left caudate head. MoCA was independently associated with left caudate head connectivity to the left OP/LG (cluster 1), precuneus/SOC (cluster 2), and the left IOC (cluster 3); B) Seed: Right caudate head. MoCA was independently associated with right caudate head connectivity to the precuneus/SOC (cluster 1), and the right OP/IOC/FG (cluster 2). Panels (left to right) represent: (1) 3-D volume rendering of significant clusters; (2) selected axial and sagittal slices for visualization; and (3) extracted relationship between MBI-C and connectivity for each cluster *(see*Table 3*for cluster details)*.

We performed a supplementary analysis assessing the relationship between right and left caudate head, and left dorsal putamen connectivity with MoCA scores, while adjusting for MBI-C and UPDRS-III. This analysis revealed no significant clusters at *p* < 0.001, cluster *p* < 0.05 FDR corrected. However, given the supplementary nature of this analysis, we explored for potential effects further by slightly lowering the height threshold to *p* < 0.005, while maintaining the cluster *p* < 0.05 FDR corrected threshold (Table 3). This revealed a significant relationship between MoCA scores and increased connectivity of both the left (Fig. 6A) and right (Fig. 6B) caudate head to the precuneus and occipital cortex. Even at this lower threshold, there was no relationship between MoCA and dorsal putamen connectivity.

**Table 3:** Significant clusters from seed-based analysis assessing the relationship of striatal connectivity with MoCA. Analysis was adjusted for MBI-C and UPDRS-III.

Seed	Location	MNI (x,y,z)	Size (voxels)	Peak p value	Cluster p value (FDR)
Left Caudate Head					
Cluster 1	Left OP/LG	−12, −80, 02	539	0.000003	0.000076
Cluster 2	Precuneus/SOC	−20, −66, 44	183	0.000141	0.0374
Cluster 3	Left IOC	−40, −72, 02	183	0.000108	0.0374
Right Caudate Head					
Cluster 1	Precuneus/SOC	−08, −66, 60	259	0.000036	0.0202
Cluster 2	Right OP/IOC/FG	16, −92, −10	211	0.000042	0.0305

*OP = occipital pole; LG = lingual gyrus; IOC = inferior occipital cortex; SOC = superior occipital cortex; FG = fusiform gyrus*.

*p < 0.005, cluster p < 0.05 FDR corrected.

## 4 Discussion

We investigated for the first time the relationship between MBI and corticostriatal connectivity in PD.

### 4.1 Relationship between MBI-C and cognition

The relationship between MBI-C and cognitive ability from a subset of these subjects has previously been reported (Yoon et al., 2019). Subjects with PD-MBI had worse cognitive abilities in all domains when compared to HC and had lower z-scores in MoCA, attention, visuospatial ability, and memory as compared to PD-noMBI.

### 4.2 Atlas-based analysis

From a network perspective, we observed reduced connectivity between the striatum and the DMN in PD-MBI. Connectivity between the DMN and caudate was previously shown to be decreased in PD patients compared to healthy controls (van Eimeren et al., 2009), and has been shown to be related to untreated depression (Bluhm et al., 2009), and psychosis risk (Hua et al., 2019) in otherwise healthy subjects. Further, increased striatal-DMN connectivity was associated with better performance on a task requiring cognitive flexibility in healthy subjects (Vatansever et al., 2015). These results suggest striatal-DMN connectivity plays a role in behavioral and cognitive phenomena, and may be altered in PD. Our results are consistent with this idea, specifically implicating striatal-DMN decoupling in the pathophysiology of MBI in PD.

Striatal connectivity to the SAN was also decreased in PD-MBI vs HC, and in PD-MBI vs PD-noMBI, though it was not significantly different in PD-noMBI vs HC. Striatal-SAN coupling has previously been shown to be related to disease severity, with less connectivity associated with increased disease severity (Putcha et al., 2015). In that study, disease severity was measured with the total UPDRS score, which takes into account each of cognitive, behavioral, motor, and functional impairments. Our results are consistent with this finding. Importantly, our results also suggest that the reduced striatal-SAN connectivity is partly accounted for by increased atrophy within the SAN in PD-MBI subjects.

### 4.3 Seed-based analysis

Next, we assessed the relationship between MBI-C scores with the connectivity of specific sub-regions of the striatum in order to gain improved spatial resolution. This analysis revealed a relationship between global behavioral impairment and connectivity of the right and left caudate head, as well as connectivity of the left dorsal putamen. Greater MBI burden was related to decreased connectivity of the left caudate head with the dorsal anterior cingulate and the left middle frontal gyrus. Greater MBI burden was also related to decreased connectivity of the left dorsal putamen and the left inferior temporal gyrus. Lastly, there were strong relationships between MBI-C and right caudate head connectivity. Decreased connectivity to the precuneus/superior occipital cortex, dorsal anterior cingulate, supramarginal/angular gyrus, and precentral gyrus was related with worse behavioral scores. Meanwhile, increased connectivity of the right caudate head with the posterior hippocampus and right cerebellar hemisphere was also related to higher MBI-C scores. Overall, this pattern of connectivity is consistent with dysfunction in the associative and limbic striatal loops, long associated with cognitive and emotional functionality (Alexander et al., 1986). In particular, caudate connectivity to the dorsal anterior cingulate cortex was strongly related to MBI-C, and this relationship was seen for both the right and left caudate head. The dACC has been implicated in broad range of cognitive and emotional functions, including reward-based decision making (Bush et al., 2002), fear expression (Milad et al., 2007), behavioral adaptation (Sheth et al., 2012), and cognitive valuation and control (Shenhav et al., 2016). Importantly, its connections with the striatum have been proposed to be central for maintaining normal motivated behavior, and dysfunction of the striatal-dACC pathway is central to the neurobiology of apathy across disease conditions, including in PD (Le Heron et al., 2018). The finding of increased connectivity between the caudate head to the hippocampus has previously been described in subjects with MCI (without PD) when compared to HC (Wang et al., 2011). The authors of that study speculated this may represent a mechanism aimed at recruiting additional network resources to compensate for neurodegenerative changes. A similar compensatory hypothesis may explain the increased connectivity seen in the present study.

Importantly, all of the seed-based analyses were adjusted for MoCA and UPDRS-III. This suggests that the distributed connectivity of the striatum, and in particular the head of the caudate and dorsal putamen, contribute to behavioural impairment in Parkinson's disease independently from cognitive ability or motor severity. However, given the relationship of behavioral and cognitive impairment, we were interested in assessing if there was some overlap in the neural representation. Indeed, connectivity of both the right and left caudate head was also related to global cognitive ability, while controlling for MBI-C and UPDRS-III. Caudate head connectivity therefore represents a component of the hypothesized shared neural representation of global behavioral and cognitive impairment in PD (O'Callaghan et al., 2014). In particular, right caudate head connectivity to clusters within the precuneus/superior occipital cortex were independently related to both MBI-C and MoCA, perhaps suggesting particular importance of this pathway. The precuneus, as a key node of the DMN (Utevsky et al., 2014), is involved in self-relational processing, episodic memory retrieval, visuospatial imagery, and consciousness (Cavanna and Trimble, 2006). Along with having extensive structural connections with distributed regions of associative cortex, the precuneus also has major connections to regions of the striatum including the dorsolateral caudate and putamen (Yeterian and Pandya, 1993). Striatal-precuneus connectivity was found to be decreased in PD compared to HC, and was shown to be related to cognitive ability (Anderkova et al., 2017). In contrast to our findings, that study found a negative correlation between striatal-precuneus connectivity and cognitive ability: patients with the worst deficits had connectivity levels close to healthy controls. The reason for this discrepancy in the direction of the relationship between connectivity and cognition is unclear. Nevertheless, our results further implicate caudate-precuneus connectivity not only in cognitive decline, but also in global behavioral impairment in PD. This is consistent with the previously cited literature showing striatal-DMN coupling is related to a range of NPS in non-PD populations (Bluhm et al., 2009; Hua et al., 2019).

### 4.4 Limitations

Firstly, our sample size was too small to examine the specific domains of MBI. Each domain might have its own unique association with cognition and its own unique neural representation. Cognitive impairment in PD consists of at least two dimensions, which likely have different pathological mechanisms and are associated with distinct neural networks (Kehagia et al., 2012; Williams-Gray et al., 2009). The dysexecutive dimension is associated with dopaminergic dependant frontostriatal connectivity (Kehagia et al., 2012; Williams-Gray et al., 2009), as well as connectivity within the sensorimotor network (Lang et al., 2019). The posterior cortical dimension is associated with cholinergic dysfunction (Kehagia et al., 2012), as well as abnormal connectivity (Lang et al., 2019) and atrophy of the temporal lobes (Burton et al., 2004). The relationship between these two cognitive dimensions and MBI in PD has yet to be determined. However, one clue towards this relationship comes from work showing increased prevalence of NPS in patients with amnestic mild cognitive impairment (Monastero et al., 2013). Future work should examine the relationship of subtypes of cognitive impairment with subtypes of MBI, and the underlying neural representations.

Lastly, impulse control symptoms in PD may be the result of medication over-dose or side-effects, rather than symptoms of the neurodegenerative process. In particular, dopamine agonist use in PD has a dose-effect relationship with impulse control disorders: increasing dose and length of treatment are associated with increased impulse control symptoms (Corvol et al., 2018). We do not believe this is a major issue for the present investigation because there was no significant difference in LED between PD-MBI and PD-noMBI, and the largest contributor towards the global MBI-C score was the Mood/Anxiety subdomain.

## 5 Conclusion

MBI, as measured with the MBI-C, represents a global marker of NPS and is significantly related to cognitive ability in PD. In the present study, altered connectivity between the striatal network and both the DMN and SAN was seen in PD-MBI. In the case of striatal-SAN connectivity, the observed reduction resulted partly from increased cortical atrophy of the SAN. Further, MBI-C scores were specifically related to the distributed connectivity of the bilateral caudate head and left dorsal putamen to regions such as the dorsal anterior cingulate cortex, the temporal cortex, the precuneus, and the occipital cortex. These regions are significant nodes in the SAN and DMN, and contribute to the limbic and associative striatal loops. Lastly, connectivity of the bilateral caudate head to the precuneus/superior occipital cortex was related to both global behavioral and cognitive scores.

In sum, our results suggest that a combination of dysfunctional striatal interactions with cortical networks (such as the DMN), and increased cortical atrophy (within the SAN), are important in the pathophysiology of global behavioral impairment in PD. Specifically, precuneus-caudate connectivity may represent a shared neural underpinning of global behavioural and cognitive impairment. This connection might therefore be of particular relevance for the relationship between MBI and subsequent cognitive decline and dementia. Further work assessing the domains of the MBI-C will need to be performed to understand if these results are truly driven by global behavioral dysfunction, or if they result from individual MBI domains. Importantly, longitudinal studies should assess whether high MBI-C scores are associated with the progression to dementia in PD, and if the connectivity profiles identified here can be used to predict and/or track this progression.

## Funding

This work was funded by a project grant from the Canadian Institutes of Health Research (CIHR) (PJT-166123), the Tourmaline Oil Chair in Parkinson's Disease, the Canada Research Chair in non-motor symptoms of Parkinson's disease to OM.

## CRediT authorship contribution statement

**Stefan Lang:** Conceptualization, Methodology, Investigation, Formal analysis, Writing - original draft, Writing - review & editing, Visualization. **Eun Jin Yoon:** Formal analysis, Writing - review & editing. **Mekale Kibreab:** Investigation, Data curation. **Iris Kathol:** Investigation, Project administration, Data curation. **Jenelle Cheetham:** Investigation, Project administration, Data curation. **Tracy Hammer:** Investigation, Data curation. **Justyna Sarna:** Investigation. **Zahinoor Ismail:** Conceptualization, Writing - review & editing, Supervision. **Oury Monchi:** Conceptualization, Methodology, Writing - review & editing, Supervision.

## Declaration of Competing Interest

There are no conflicts of interest to declare for any author.

## Appendix Supplementary materials

Image, application 1

Image, application 2

Image, application 3

Image, application 4

Image, application 5
