# Supplementary material for: Mild behavioral impairment in Parkinson's disease is associated with altered corticostriatal connectivity
Source: Neuroimage Clin. 2020 Mar 27;26:102252. doi: 10.1016/j.nicl.2020.102252 (PMC7152681; doi:10.1016/j.nicl.2020.102252)
Supplement: Supplementary file 1 [file mmc1.docx]

**Supplementary Methods I: MRI Acquisition and Analysis**

**Mild behavioural impairment in Parkinson’s disease is associated with altered corticostriatal connectivity**

Stefan Lang^1,2,3^, Eun Jin Yoon^1,2,3^, Mekale Kibreab^1^, Iris Kathol^1^, Jenelle Cheetham^1^, Tracy Hammer^1^, Justyna Sarna^1,2,3^, Zahinoor Ismail^1,2,3,4,5^, Oury Monchi ^1,2,3,6^

1 Cumming School of Medicine, University of Calgary, Calgary, AB, CA;

2 Department of Clinical Neuroscience, University of Calgary, Calgary, AB, CA

3 Hotchkiss Brain Institute, University of Calgary, Calgary, AB, CA;

4 Department of Psychiatry, University of Calgary, Calgary, AB, CA;

5 Mathison Center for Brain and Mental Health Research, University of Calgary, Calgary, CA;

6 Department of Radiology, University of Calgary, Calgary, AB, CA;

1. *MRI Acquisition and Analysis*

Subjects were scanned at the Seaman Family MR Center, at the University of Calgary, with a 3T GE Discovery MR750 scanner. Sessions included a high-resolution, T1-weighted, 3D volume acquisition for anatomic localization (TR=7.18 ms, TE=2.25 ms, flip angle 10^o^, voxel size 1mm^3^, 172 slices), followed by echo-planar T2*-weighted image acquisitions with BOLD contrast (TR=2.9 sec, echo time=30 ms; flip angle, 90^o^, voxel size 2.5mmx2.5mmx3mm, 48 slices, 152 volumes). Resting-state fMRI was acquired over 1 run in a single session. During the scan, participants were presented with a black fixation cross on a white background, and were instructed to keep their eyes open and look at the cross.

1. *Image Pre-Processing*

Images were preprocessed using SPM 12[1]. Briefly, functional images underwent realignment and unwarping as well as slice-time correction. The high-resolution structural images were co-registered to the mean functional image. Visual inspection for quality control was performed at each stage. The co-registered structural images were segmented into grey matter, white matter and cerebrospinal fluid (CSF). Functional images were non-linearly normalized into MNI space using SPM unified normalization[2]. No spatial smoothing was performed, in accordance with recent literature suggesting this preprocessing step can artificially increase the similarity of networks across subjects[3]. Images were spatially resampled at 2 mm^3^ prior to analysis.

1. *Image Denoising*

Denoising of the functional data was performed using the MATLAB toolbox Conn[4]. Physiological and other sources of noise from the white matter and CSF signal were estimated using the aCompcor method [5,6]. Five principle components were extracted from eroded CSF and white matter masks, and included as covariates of no-interest. To account for motion, movement parameters, and their first temporal derivative, were also included in the regression. Further quality assurance to detect outliers in motion and global signal intensity change was performed. Volumes with greater than 3 mm change of maximal composite motion, or a blood oxygen level dependent (BOLD) change > 3 SD from the mean, were flagged and included as regressors in the first level analysis. Linear detrending, to remove signal drift, was performed. The residual BOLD time series was subjected to a high-pass filter (>0.008 Hz) prior to calculation of resting state connectivity. A full band pass filter (i.e 0.008-0.1Hz) was not used, as there is accumulating evidence for the relevance of higher frequencies in the resting state signal[7].

**References**

[1] K. Friston, Statistical Parametric Mapping: The analysis of functional brain images, Academic Press, London, 2007. https://doi.org/https://doi.org/10.1016/B978-012372560-8/50001-2.

[2] J. Ashburner, K.J. Friston, Unified segmentation, Neuroimage. 26 (2005) 839–851. https://doi.org/https://doi.org/10.1016/j.neuroimage.2005.02.018.

[3] T. Alakörkkö, H. Saarimäki, E. Glerean, J. Saramäki, O. Korhonen, Effects of spatial smoothing on functional brain networks, Eur. J. Neurosci. 46 (2017) 2471–2480. https://doi.org/10.1111/ejn.13717.

[4] S. Whitfield-Gabrieli, A. Nieto-Castanon, Conn: a functional connectivity toolbox for correlated and anticorrelated brain networks, Brain Connect. 2 (2012) 125–141. https://doi.org/10.1089/brain.2012.0073 [doi].

[5] Y. Behzadi, K. Restom, J. Liau, T.T. Liu, A component based noise correction method (CompCor) for BOLD and perfusion based fMRI, Neuroimage. 37 (2007) 90–101. https://doi.org/S1053-8119(07)00383-7 [pii].

[6] X.J. Chai, A.N. Castanon, D. Ongur, S. Whitfield-Gabrieli, Anticorrelations in resting state networks without global signal regression, Neuroimage. 59 (2012) 1420–1428. https://doi.org/10.1016/j.neuroimage.2011.08.048 [doi].

[7] J.E. Chen, G.H. Glover, BOLD fractional contribution to resting-state functional connectivity above 0.1Hz, Neuroimage. 107 (2015) 207–218. https://doi.org/https://doi.org/10.1016/j.neuroimage.2014.12.012.
