# Supplementary material for: Mild behavioral impairment in Parkinson's disease is associated with altered corticostriatal connectivity
Source: Neuroimage Clin. 2020 Mar 27;26:102252. doi: 10.1016/j.nicl.2020.102252 (PMC7152681; doi:10.1016/j.nicl.2020.102252)
Supplement: Supplementary file 2 [file mmc2.docx]

**Supplementary Methods III: Assessment and Removal of Outliers**

**Mild behavioral impairment in Parkinson’s disease is associated with altered corticostriatal connectivity**

Stefan Lang^1,2,3^, Eun Jin Yoon^1,2,3^, Mekale Kibreab^1^, Iris Kathol^1^, Jenelle Cheetham^1^, Tracy Hammer^1^, Justyna Sarna^1,2,3^, Zahinoor Ismail^1,2,3,4,5^, Oury Monchi ^1,2,3,6^

1 Cumming School of Medicine, University of Calgary, Calgary, AB, CA;

2 Department of Clinical Neuroscience, University of Calgary, Calgary, AB, CA

3 Hotchkiss Brain Institute, University of Calgary, Calgary, AB, CA;

4 Department of Psychiatry, University of Calgary, Calgary, AB, CA;

5 Mathison Center for Brain and Mental Health Research, University of Calgary, Calgary, CA;

6 Department of Radiology, University of Calgary, Calgary, AB, CA;

Outliers in the MBI-C data were identified by performing a square-root transformation and calculating the z-score. Any data point with a z-score >3 (≥ 3 standard deviations from the mean) was classified as an outlier. This identified one subject who could be considered an outlier (Supplementary Figure 2). The seed based analysis assessing the relationship of MBI-C scores with corticostriatal connectivity was repeated as described in the main manuscript without this subject. This was adjusted for MoCA and UPDRS. Significant clusters were defined with a height threshold of p<0.001 (two-tailed, uncorrected), followed by a cluster threshold of p<0.05 with a false discovery rate (FDR) correction for multiple comparisons.

^
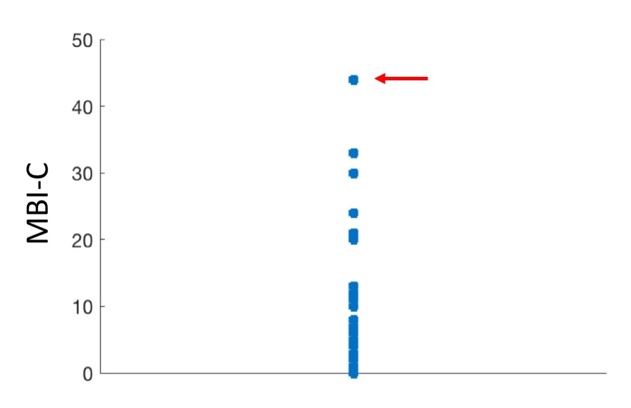
^

***Supplementary Figure 2.*** *Scatter plot of MBI-C scores (y-axis) from the Parkinson’s disease subjects. One outlier is detected (red arrow), with an MBI-C score of 44.*

Decreased connectivity between the left caudate head and the dorsal anterior cingulate cortex was still significantly associated with MBI-C scores with the outlier subject removed **(Supplementary Figure 2)**. Significant clusters were no longer observed between the left dorsal putamen and the left inferior temporal pole.

**
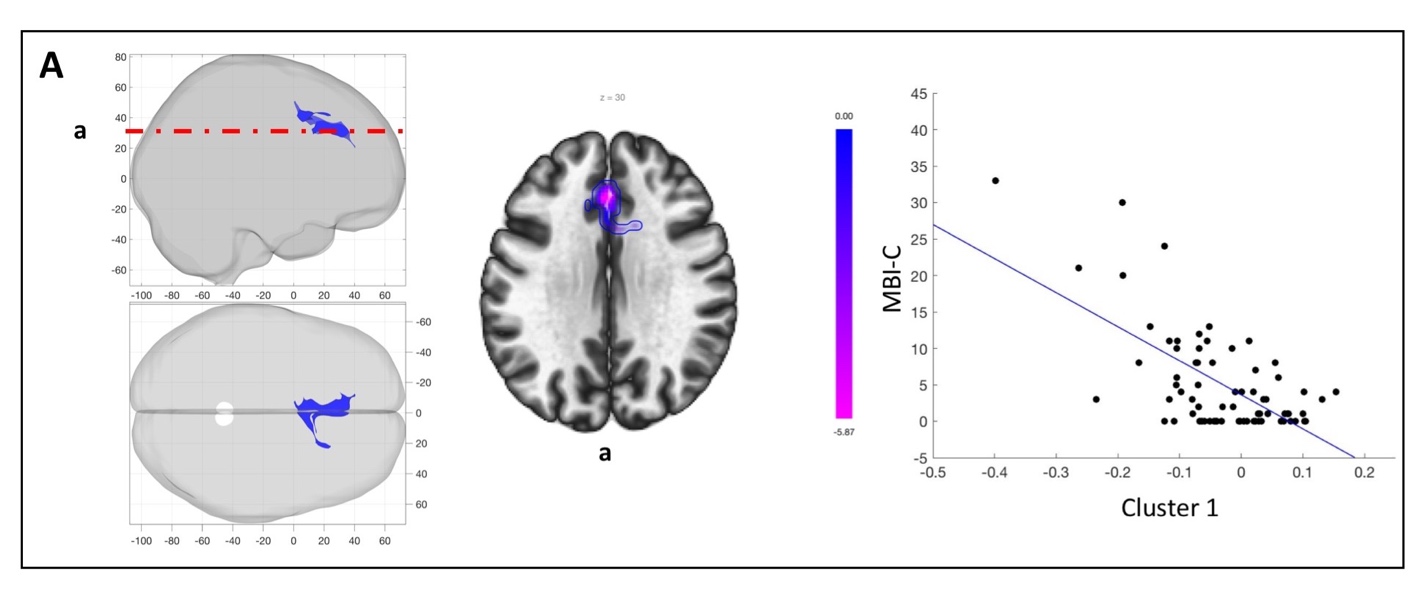
**

***Supplementary Figure 3.*** *Relationship between MBI-C and striatal subdivision connectivity, adjusting for MoCA and UPDRS-III (while removing outlier subject). Seed: Left caudate head. MBI-C was independently associated with left caudate head connectivity to the dorsal ACC (cluster 1). ). Panels (left to right) represent: (1) 3-D volume rendering of significant clusters, with MNI coordinates on the axes; (2) selected axial slice for visualization; and (3) extracted relationship between MBI-C (y-axis) and connectivity (x-axis) for each cluster (see Supplementary Table 3 for cluster details).*

With respect to the right caudate head seed, clusters remained significant in the dorsal anterior cingulate, precuneus/superior occipital cortex, and right cerebellum. Further, a cluster emerged in the right middle/superior frontal gyrus **(Supplementary Figure 3)**. All cluster details from this supplementary analysis are reported in **Supplementary Table 3**.


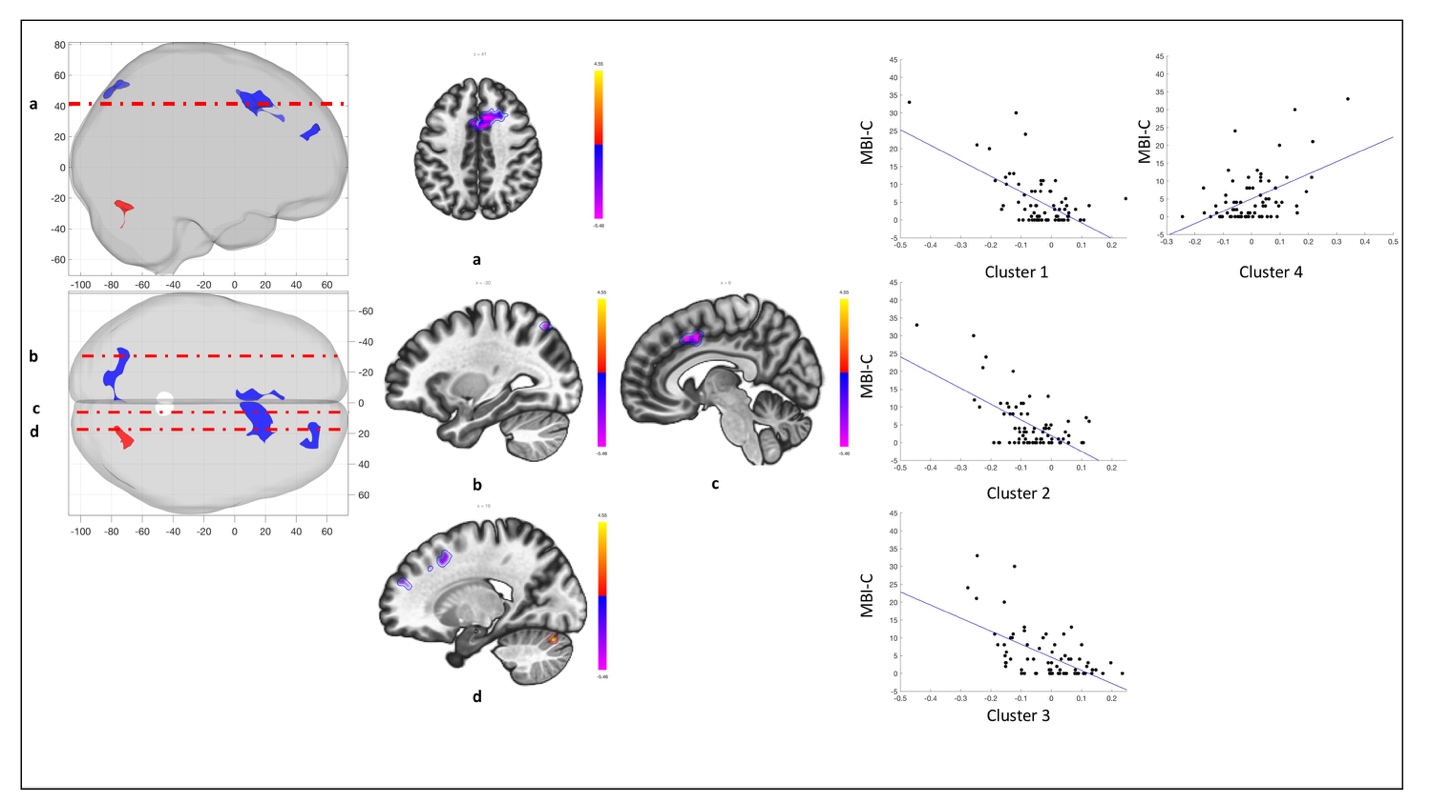


***Supplementary Figure 4.*** *Relationship between MBI-C and striatal subdivision connectivity, adjusting for MoCA and UPDRS-III (while removing outlier subject). Seed: Right caudate head. MBI-C was independently associated with right caudate head connectivity to the dorsal ACC (cluster 1), precuneus/superior occipital cortex (cluster 2), middle/superior frontal gyrus (cluster 3) and right cerebellum (cluster 4). Panels (left to right) represent: (1) 3-D volume rendering of significant clusters, with MNI coordinates on the axes; (2) selected axial slice for visualization; and (3) extracted relationship between MBI-C (y-axis) and connectivity (x-axis) for each cluster (see Supplementary Table 3 for cluster details).*

**Supplementary Table 3. Significant clusters from the seed-based analysis assessing the relationship of striatal connectivity with MBI-C scores, after outlier removal. Analysis was adjusted for MoCA and UPDRS-III.**

| **Seed** | **Location** | **MNI (x,y,z)** | **Size (voxels)** | **Peak p value** | **Cluster p value (FDR)** |
| --- | --- | --- | --- | --- | --- |
| **Left Caudate Head** |  |  |  |  |  |
| Cluster 1 | Dorsal ACC | 04, 30, 32 | 624 | <0.0000001 | <0.0000001 |
| **Left Dorsal Putamen** |  |  |  |  |  |
| - | - | - | - | - | - |
| **Right Caudate Head** |  |  |  |  |  |
| Cluster 1 | Dorsal ACC | 06, 08, 46 | 649 | 0.000001 | <0.0000001 |
| Cluster 2 | Precuneus/SOC | -14, -84, 46 | 262 | 0.000001 | 0.000127 |
| Cluster 3 | Right SFG/MFG | 18, 54, 26 | 156 | 0.000032 | 0.00322 |
| Cluster 4 | Right Cerebellum | 18, -76, -24 | 139 | 0.000023 | 0.004638 |

**p<0.001, cluster p<0.05 FDR corrected*

*ACC = anterior cingulate cortex; SFG = superior frontal gyrus; MFG = middle frontal gyrus; SOC = superior occipital cortex;*
