# Supplementary material for: Mild behavioral impairment in Parkinson's disease is associated with altered corticostriatal connectivity
Source: Neuroimage Clin. 2020 Mar 27;26:102252. doi: 10.1016/j.nicl.2020.102252 (PMC7152681; doi:10.1016/j.nicl.2020.102252)
Supplement: Supplementary file 3 [file mmc3.docx]

**Supplementary Methods II: Structural Analysis**

**Mild behavioral impairment in Parkinson’s disease is associated with altered corticostriatal connectivity**

Stefan Lang^1,2,3^, Eun Jin Yoon^1,2,3^, Mekale Kibreab^1^, Iris Kathol^1^, Jenelle Cheetham^1^, Tracy Hammer^1^, Justyna Sarna^1,2,3^, Zahinoor Ismail^1,2,3,4,5^, Oury Monchi ^1,2,3,6^

1 Cumming School of Medicine, University of Calgary, Calgary, AB, CA;

2 Department of Clinical Neuroscience, University of Calgary, Calgary, AB, CA

3 Hotchkiss Brain Institute, University of Calgary, Calgary, AB, CA;

4 Department of Psychiatry, University of Calgary, Calgary, AB, CA;

5 Mathison Center for Brain and Mental Health Research, University of Calgary, Calgary, CA;

6 Department of Radiology, University of Calgary, Calgary, AB, CA;

Two high-resolution T1-weighted images of 3D inversion recovery fast spoiled prepared gradient recalled sequence were acquired for each patient with the GE DISCOVERY MR750 3.0T MRI at the Seaman Family Imaging Centre at the University of Calgary (TR=7.18 ms, TE=2.25 ms, flip angle 10^o^, voxel size 1mm^3^, 172 slices). The Free-Surfer imaging analysis suite (version 6.0.0, surfer.nmr.mgh. harvard.edu/) was used to estimate cortical thickness and subcortical volume. The details of these procedures have been extensively described in prior publications(Fischl and Dale, 2000), and are the same as those in an earlier publication on MBI in PD(Yoon et al., 2019) Briefly, two volume images underwent motion correction and averaging to enhance the gray-to-white contrast to noise, skull stripping, transform to Talairach space, segmentation of subcortical white and gray matter structures, intensity normalization, tessellation of the gray/white matter boundaries, automated topology correction, and surface deformation following intensity gradients to optimally place the gray/white (white matter surface) and gray/CSF (pial surface) borders that most accurately define the transition to the other tissue class. Segmented volumes were visually inspected, and the appropriate manual corrections were performed. The local cortical thickness was measured on the basis of the difference between the position of equivalent vertices in the pial and white matter surfaces. Each face of the white surface and its matching face in the pial surface are used to define an oblique truncated pyramid; then cortical volume is computed as the volume of a truncated tetrahedron. We calculated the average thickness (measured in mm) within the cortical networks (CEN, SAN and DMN) by averaging the thickness value within each ROI of each network. We calculated the average volume of the striatal network by averaging the volume of each ROI within that network. We also segmented and calculated the volume of the striatal subdivisions used in the seed based analysis. All measurements of cortical volume were normalized by the total intracranial volume.

One subject (PD-noMBI) had only one structural scan, and this did not have sufficient quality of to accurately perform the analysis. This left a total N of 101 (28 HC; 21 PD-MBI; and 52 PD no-MBI). Further, the thickness estimation of three ROI’s (all within the DMN: left and right frontal pole, ventromedial prefrontal cortex) was not successful, likely due to the small size of these ROI’s. These ROI’s were left out of the following structural analysis results.

Normality of the structural data was assessed with Kolmogorov-Smirnov, which was not significant. Group differences in average network atrophy between PD-MBI, PD-noMBI, and HC were evaluated with Welch’s ANOVA and post-hoc Games-Howell tests, as in the main analysis of functional connectivity differences. The group comparison of functional connectivity was then repeated while adjusting for any atrophy values which were significantly different.

We also correlated the MBI-C score with each of the striatal sub-division volumes. This was done to determine if this structural measure could be responsible for the relationship between the connectivity of each region and the MBI-C. If the striatal volume was significantly related to MBI-C scores, this would be used as a covariate in the seed-based analysis of functional connectivity.

We observed a significant group difference in the thickness of the SAN (F(1,43.75)=7.74, p=0.0079). Post-hoc tests revealed this was due to increased average atrophy of the SAN in PD-MBI vs PD-noMBI (p=0.0221) and PD-MBI vs HC (p=0.0039). There was no group difference in the atrophy pattern of the DMN (F(1,34.17)=1.56, p=0.2204), the CEN (F(1,39.57)=0.060, p=0.8072), or the striatal network (F(1,34.10)=1.96, p=0.1706). Given the significant difference in atrophy within the SAN, we repeated the group comparison of functional connectivity between the striatal network and the SAN, while adjusting for average atrophy within the SAN. Once SAN atrophy was adjusted for, the group difference in functional connectivity was no longer statistically significant with correction for multiple comparison (F(1,52.40)=4.97, p=0.0301).


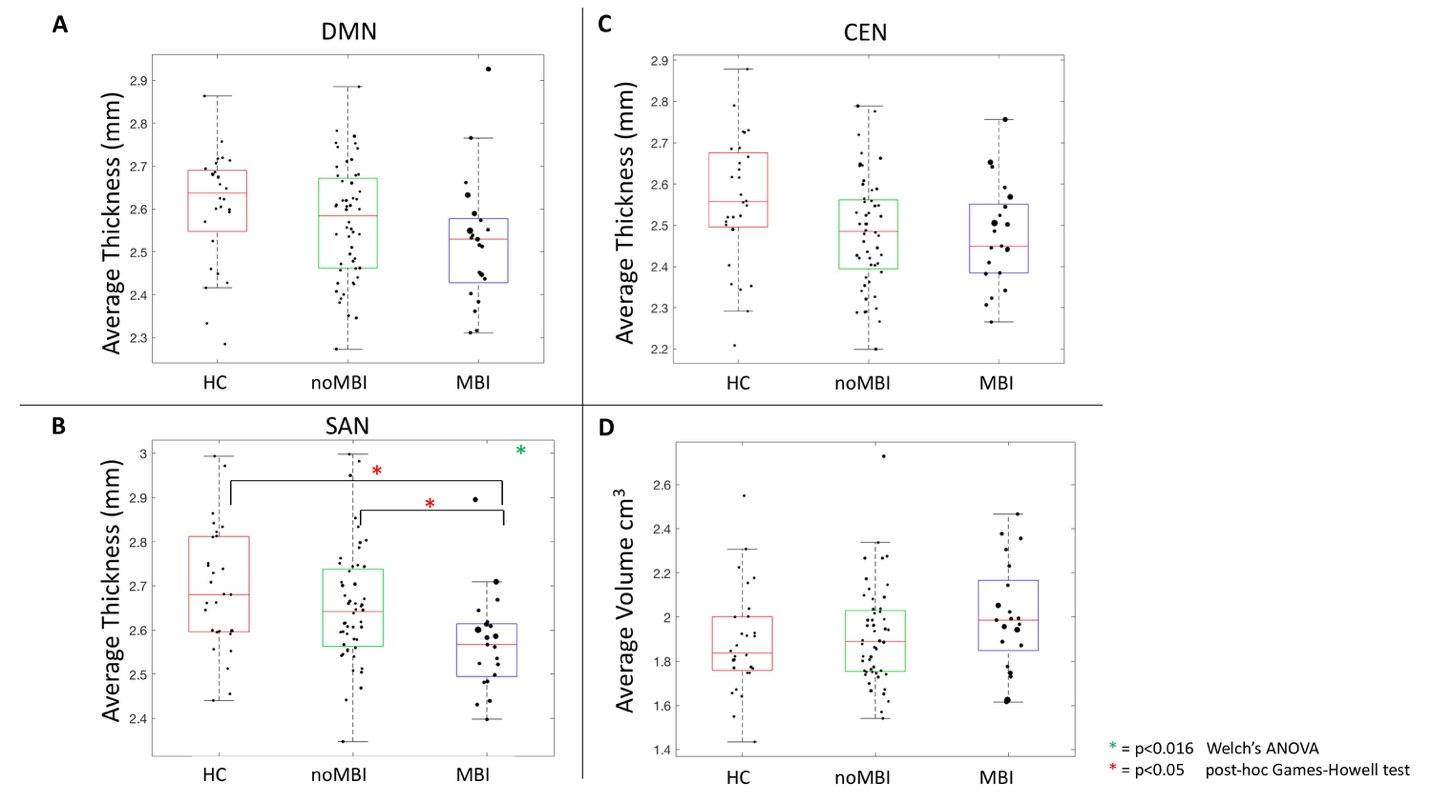


***Supplementary Figure 1.*** *Group differences in average thickness and volume of the networks used in the Atlas based analysis. A) DMN thickness; B)SAN thickness; C) CEN thickness. Welch’s ANOVA with significance set at p<0.0167. Post-hoc comparison using Games-Howell test with significance set at p<0.05. For visualization, the diameter of each data point is representative of the MBI-C score: large data points equal higher scores.*

With respect to the seed based analysis, none of the relevant seeds showed a relationship between local volume and MBI-C scores (left caudate head: r^2^ = 0.0061, p = 0.5105; right caudate head: r^2^ = 0.0003, p = 0.8773 ; left dorsal putamen: r^2^ = 0.0111, p = 0.3759 ). The seed-based analysis of functional connectivity was therefore not adjusted for with the volumetric measurements.

**References:**

Fischl, B., Dale, A.M., 2000. Measuring the thickness of the human cerebral cortex from magnetic resonance images. Proc. Natl. Acad. Sci. 97, 11050 LP – 11055. https://doi.org/10.1073/pnas.200033797

Yoon, E.J., Ismail, Z., Hanganu, A., Kibreab, M., Hammer, T., Cheetham, J., Kathol, I., Sarna, J.R., Martino, D., Furtado, S., Monchi, O., 2019. Mild behavioral impairment is linked to worse cognition and brain atrophy in Parkinson disease. Neurology 93, e766 LP-e777. https://doi.org/10.1212/WNL.0000000000007968
