# Supplementary material for: Mild behavioral impairment in Parkinson's disease is associated with altered corticostriatal connectivity
Source: Neuroimage Clin. 2020 Mar 27;26:102252. doi: 10.1016/j.nicl.2020.102252 (PMC7152681; doi:10.1016/j.nicl.2020.102252)
Supplement: Supplementary file 4 [file mmc4.docx]

**Mild behavioural impairment in Parkinson’s disease is associated with altered corticostriatal connectivity**

Stefan Lang^1,2,3^, Eun Jin Yoon^1,2,3^, Mekale Kibreab^1^, Iris Kathol^1^, Jenelle Cheetham^1^, Tracy Hammer^1^, Justyna Sarna^1,2,3^, Zahinoor Ismail^1,2,3,4,5^, Oury Monchi ^1,2,3,6^

1 Cumming School of Medicine, University of Calgary, Calgary, AB, CA;

2 Department of Clinical Neuroscience, University of Calgary, Calgary, AB, CA

3 Hotchkiss Brain Institute, University of Calgary, Calgary, AB, CA;

4 Department of Psychiatry, University of Calgary, Calgary, AB, CA;

5 Mathison Center for Brain and Mental Health Research, University of Calgary, Calgary, CA;

6 Department of Radiology, University of Calgary, Calgary, AB, CA;

**Supplementary Table 1:** **Neuropsychological Tests grouped into five cognitive domains**

| **Global Cognition** |  |
| --- | --- |
|  | MOCA |
| **Executive Function** |  |
|  | STROOP Colour-Word |
|  | Brixton Spatial Anticipation |
|  | Hayling Sentence Completion |
|  | Trail Making Test (B) |
|  | Clock Drawing Test (Command) |
| **Attention** |  |
|  | Trail Making Test (A) |
|  | WMS-IV Symbol Span |
|  | WAIS-IV Digit Span (FWD) |
| **Language** |  |
|  | Boston Naming Test |
|  | Semantic Fluency (Animals/Actions) |
| **Visuospatial** |  |
|  | HVOT/Benton JOLO* |
|  | RCFT Copy trial |
| **Memory** |  |
|  | HVLT |
|  | WMS-IV Logical Memory |
|  | RCFT recall trials |

MOCA: Montreal Cognitive Assessment; DRS: Hooper Visual Organization Test; JOLO: Judgement of Line Orientation; RCFT: Rey Complex Figure Copy; HVLT: Hopkins Verbal Learning Test

* Twenty-four Parkinson’s disease participants were administered the Benton Judgement of Line Orientation instead of the Hooper Visual Organization Test. Both tests have been previously used to assess visuospatial abilities in Parkinson’s disease patients (1) and are significantly correlated with each other (2).

**References**

1. Lawrence BJ, Gasson N, Loftus AM. Prevalence and Subtypes of Mild Cognitive Impairment in Parkinson’s Disease. Sci Rep. 2016;1–9.

2. Silva Pena CM, Sobreira STE, Souza CP, Oliveria NG, Tumas V, do Vale de ACF. Visuospatial cognitive tests for the evaluation of patients with Parkinson’s disease. Dement Neuropsycholgia. 2008;2(3):201–5.
