# Supplementary material for: Mild behavioral impairment in Parkinson's disease is associated with altered corticostriatal connectivity
Source: Neuroimage Clin. 2020 Mar 27;26:102252. doi: 10.1016/j.nicl.2020.102252 (PMC7152681; doi:10.1016/j.nicl.2020.102252)
Supplement: Supplementary file 5 [file mmc5.docx]

**Mild behavioural impairment in Parkinson’s disease is associated with altered corticostriatal connectivity**

Stefan Lang^1,2,3^, Eun Jin Yoon^1,2,3^, Mekale Kibreab^1^, Iris Kathol^1^, Jenelle Cheetham^1^, Tracy Hammer^1^, Justyna Sarna^1,2,3^, Zahinoor Ismail^1,2,3,4,5^, Oury Monchi ^1,2,3,6^

1 Cumming School of Medicine, University of Calgary, Calgary, AB, CA;

2 Department of Clinical Neuroscience, University of Calgary, Calgary, AB, CA

3 Hotchkiss Brain Institute, University of Calgary, Calgary, AB, CA;

4 Department of Psychiatry, University of Calgary, Calgary, AB, CA;

5 Mathison Center for Brain and Mental Health Research, University of Calgary, Calgary, CA;

6 Department of Radiology, University of Calgary, Calgary, AB, CA;

**Supplementary Table 2: Region of Interest details**

| **ROI#** | **Anatomical Region** | **Network** | **Component #, MNI coordinates, voxel size** |
| --- | --- | --- | --- |
| **1** | Left Inferior Occipital-Temporal (lateral) | CEN L | ICA_18+ (-51,-51,-19) n=165 |
| **2** | Right middle frontal gyrus | CEN R | ICA_21+ (36,41,28) n=1166 |
| **3** | Right lateral frontal pole | CEN R | ICA_21- (47,46,-5) n=103 |
| **4** | Right Inferior parietal | CEN R | ICA_22+ (46,-49,45) n=1790 |
| **5** | Right frontal Pole | CEN R | ICA_28+ (35,54,7) n=1574 |
| **6** | Left frontal Pole | CEN R | ICA_28+ (-36,55,6) n=327 |
| **7** | Left Angular gyrus | CEN L | ICA_36+ (-44,-58,44) n=1716 |
| **8** | Right inferior frontal gyrus | CEN R | ICA_40+ (53,29,12) n=1738 |
| **9** | Medial prefrontal cortex(dorsal) | CEN R & L | ICA_43+ (3,31,55) n=2258 |
| **10** | Left Insula (superior) | SAN Anterior | ICA_13+ (-40,1,10) n=674 |
| **11** | Right Insula (superior) | SAN Anterior | ICA_13+ (41,1,10) n=440 |
| **12** | Left Insula (ventral) | SAN Anterior/Posterior | ICA_26+ (-44,4,-8) n=916 |
| **13** | Right insula (ventral) | SAN Anterior/Posterior | ICA_26+ (44,6,-10) n=725 |
| **14** | Left supra-marginal gyrus | SAN Posterior | ICA_34+ (-60,-29,32) n=753 |
| **15** | Right supra-marginal gyrus | SAN Posterior | ICA_34+ (62,-27,34) n=276 |
| **16** | Dorsal anterior cingulate | SAN Anterior | ICA_53+ (-3,20,39) n=915 |
| **17** | Left anterior MFG | SAN Anterior | ICA_64+ (-39,47,20) n=1680 |
| **18** | Right Heschls | SAN Posterior | ICA_65+ (47,-24,22) n=225 |
| **19** | Posterior cingulate cortex | DMN/Precuneus | ICA_10+ (1,-55,33) n=1971 |
| **20** | Anterior Cingulate Cortex | DMN dorsal | ICA_14+ (0,45,4) n=930 |
| **21** | Left frontal Pole | DMN dorsal | ICA_14- (-14,69,15) n=31 |
| **22** | Right frontal Pole | DMN dorsal | ICA_14- (14,69,14) n=67 |
| **23** | Posterior cingulate cortex | DMN dorsal | ICA_15+ (-1,-53,24) n=153 |
| **24** | Bilateral medial prefrontal cortex | DMN dorsal | ICA_29+ (-1,52,16) n=1772 |
| **25** | Bilateral medial prefrontal cortex (dorsal) | DMN dorsal | ICA_38+ (2,54,30) n=2213 |
| **26** | Ventromedial prefrontal cortex | DMN dorsal | ICA_39+ (2,35,-18) n=26 |
| **27** | Superior precuneus | DMN Ventral | ICA_47+ (-1,-57,54) n=1718 |
| **28** | Bilateral medial prefrontal cortex (dorsal) | DMN dorsal | ICA_52+ (1,37,43) n=976 |
| **29** | Left mid occipital | DMN Ventral | ICA_62+ (-38,-81,34) n=587 |
| **30** | Right mid occipital | DMN Ventral | ICA_62+ (41,-77,36) n=113 |
| **31** | left putamen | Striatal | ICA_27+ (-26,5,0) n=211 |
| **32** | right putamen | Striatal | ICA_27+ (27,6,0) n=125 |
| **33** | right dorsal caudate | Striatal | ICA_27+ (17,6,18) n=63 |
| **34** | bilateral caudate | Striatal | ICA_89+ (-1,-3,11) n=1798 |
